# An Experimental Urban Case Study with Various Data Sources and a Model for Traffic Estimation

**DOI:** 10.3390/s22010144

**Published:** 2021-12-26

**Authors:** Alexander Genser, Noel Hautle, Michail Makridis, Anastasios Kouvelas

**Affiliations:** Department of Civil, Environmental and Geomatic Engineering, Institute for Transport Planning and Systems, ETH Zurich, CH-8093 Zurich, Switzerland; nhautle@student.ethz.ch (N.H.); michail.makridis@ivt.baug.ethz.ch (M.M.); kouvelas@ethz.ch (A.K.)

**Keywords:** urban traffic state, travel time estimation, traffic management, traffic flow, license plate detection, empirical measurements, multiple linear regression

## Abstract

A reliable estimation of the traffic state in a network is essential, as it is the input of any traffic management strategy. The idea of using the same type of sensors along large networks is not feasible; as a result, data fusion from different sources for the same location should be performed. However, the problem of estimating the traffic state alongside combining input data from multiple sensors is complex for several reasons, such as variable specifications per sensor type, different noise levels, and heterogeneous data inputs. To assess sensor accuracy and propose a fusion methodology, we organized a video measurement campaign in an urban test area in Zurich, Switzerland. The work focuses on capturing traffic conditions regarding traffic flows and travel times. The video measurements are processed (a) manually for ground truth and (b) with an algorithm for license plate recognition. Additional processing of data from established thermal imaging cameras and the Google Distance Matrix allows for evaluating the various sensors’ accuracy and robustness. Finally, we propose an estimation baseline MLR (multiple linear regression) model (5% of ground truth) that is compared to a final MLR model that fuses the 5% sample with conventional loop detector and traffic signal data. The comparison results with the ground truth demonstrate the efficiency and robustness of the proposed assessment and estimation methodology.

## 1. Introduction

An accurate derivation of fundamental traffic state variables is key for sophisticated traffic management. Urban areas especially suffer from congestion due to a higher population density, traffic lights and elevated mobility demand. In addition, other variables, traffic flow information at specific locations in the network and travel times between an origin and a destination are of great importance for traffic operators. In particular, travel times allow for the derivation of the network’s current level of service (LoS) and influence the network user’s mode and route choice. Consequently, accurate sensor technology is needed to detect vehicles when traveling through a network with a low error rate where traffic variables satisfy accuracy requirements [1]. However, urban areas are sometimes sparsely equipped with sensors, which negatively affects accuracy and increases the noise level of the results. In addition, a variety of sensor technologies used in urban environments are only suitable for deriving particular traffic variables and also differ in data resolution. For example traditional sensor technologies such as loop detectors (LD) are still one of the widely used measurement devices due to reliability and flexibility in design [2]. Although, LDs have been shown as a promising data source for traffic flow, theoretical assumptions for a unique vehicle identification are needed and a re-identification is not possible. In contrast, new technologies such as video/thermal cameras and Bluetooth/WiFi sensors not only enable accurate derivation of traffic flow, but also provide good results in measuring travel time, since unique vehicle identification based on MAC address recognition is possible.

Although numerous studies have evaluated and estimated travel times on freeways and in urban areas, only few studies have compared emerging sensor technologies to an empirical ground-truth measurement. In addition, the question still remains on how traditional sensor data can help with travel time estimation in terms of performance improvement. Therefore, this work focuses on providing traffic state representation in terms of traffic flow and travel time estimation within an urban network. We run a multi-sensor campaign in Zurich, Switzerland, including video measurements from the particular area and investigate the time series derivation of traffic flow and travel times. Consequently, we compare the following data sources: (a) thermal camera sensors data that are equipped with a WiFi interface (b) processed video data with an automated license plate recognition (ALPR) algorithm and (c) Google Distance Matrix data from the particular area. For comparative results, the video data serves for the exact determination of traffic flow and travel times, i.e., a ground-truth data set. Besides the assessment of the different data sets, we propose a simple yet efficient multiple linear regression (MLR) model to estimate travel times in a future environment with connected and automated vehicles (CAV). We create a baseline scenario with a random 5% sample of the ground-truth data that emulates data from moving sensors (e.g., from CAVs). Finally, a model that fuses moving sensor data with traditional LD and traffic signal data is proposed. The estimation results are compared to the baseline and ground-truth data.

The paper includes the following contributions: (a) an experimental campaign with multiple sensor data (thermal video, LD and traffic signal, and Google Distance Matrix); (b) sensor-based analysis for traffic state estimation in terms of traffic flow and travel times; (c) accuracy assessment of the derived time series for all sensors; (d) travel time estimation for specific origin-destinations by fusing static LD and traffic signal data with (emulated) CAV information and comparison to a baseline scenario.

The remainder of the paper is as follows: The next section highlights relevant previous works in the field of traffic flow/travel time assessment and estimation. Following this, we introduce the data processing methodology and the utilized performance metrics. The travel time estimation models with used performance metrics are introduced in the subsequent section. A description of the empirical experiment and the collected data sets is given in the succeeding section. This includes a description of the area, the performed video measurement, and a description of the other sensor sources, i.e., thermal cameras, post-processed video data with ALPR, Google Distance Matrix data, and derivation of the ground-truth data set. Afterwards, the results are presented and discussed and the paper closes with a concluding section and an outline for future work.

## 2. Related Works

As a consequence of rising mobility demand, freeways and urban areas are continuously suffering from traffic congestion. This results in smaller network throughput, lower average speeds on network links, and consequently, higher travel times [3]. The traffic management domain offers several tools that influence route choice [4] and mode choice (e.g., congestion pricing [5]), or traffic demand (e.g. user’s departure time) to tackle rising congestion problems. Nevertheless, the implementation of sophisticated traffic management policies requires reliable input data, such as traffic flow and travel time dynamic estimates. Besides, travel time estimation in urban networks is particularly challenging because of the dynamic demand, low speeds and signaling. Efforts towards accurately predicting time-to-green [6] can certainly help but until high penetration levels of AVs [7] are achieved, traffic signaling remains a challenge for accurate travel time estimation.

In particular, travel times can be measured directly by identifying a vehicle at two specific points in space with a corresponding timestamp. This is achieved with camera data and the application of ALPR algorithms that allows matching license plates or probe vehicle data. In addition, novel sensors with Bluetooth or WiFi interfaces can detect a unique MAC address of devices such as, e.g., a mobile phone [8]. An evaluation of Bluetooth sensors as a potential ground-truth data source was performed in [9]. The study compares Bluetooth sensor data collected on a freeway to vehicle probe data, and the penetration rate of the Bluetooth system is approximated with LD data. Results underline that Bluetooth sensors are a promising data source to measure accurate travel times. Nevertheless, the quality of Bluetooth sensor data heavily depends on the penetration in the system. Hence, the statement that the new sensors can be utilized as ground-truth data is questionable with a low sample rate. As [10] shows, the penetration rate of Bluetooth systems can be low and hence only detect a fraction of vehicles of an observed network. The study reports that the aggregated penetration rate of several observed segments in Delaware and Maryland, USA, fluctuates around 4%. For the derivation of the ground-truth data set, LDs and microwave sensors are utilized; i.e., no actual empirical measurement of the traffic flow or travel time was performed. The results are supported by another study in Turkey, where a penetration rate of 5% was found [11]. Another field test was conducted by [12] on a freeway in Barcelona, Spain. The work utilizes Bluetooth sensors to determine travel times and then designs a Kalman filter that allows the estimation of origin-destination pairs on freeways. The authors extend their work in [13] to urban networks with route choice.

Alongside the determination of travel times for vehicles on freeways and urban areas, the quantities are also from great interest for public transportation, non-motorized transport modes, i.e., cyclists and pedestrians. [14] develops a framework to estimate the travel times of buses in Auckland, New Zealand. The framework allows for calculating a network’s traffic state, i.e., congested or not congested with positioning data. Two models are combined, where the first model estimates travel times followed by the traffic state computed by the second model. [15] shows travel time estimation for pedestrian’s and cyclist’s travel times based on Bluetooth and WiFi MAC addresses. Validation is performed with ground-truth data.

Contrary, travel times can also be derived indirectly with traditional sensors data. Loop detectors allow the direct determination of traffic flow and an approximation of speed, which can then be used to calculate travel times. A prediction of travel times based on LD data in California is proposed by [8]. The processed data serves as input to a prediction framework, including a bottleneck identification algorithm, traffic regime clustering, a stochastic congestion map for clustered data, and an algorithm for a congestion search algorithm. A more practical approach of travel time estimation was followed by [16] with LD data. The work addresses data gaps (spatially and temporally) by applying an Exponential Moving Average (EMA) to improve the estimates. Additionally, [16] transforms the calculated Time-Mean-Speed (TMS) to Space-mean-speed (SMS) by fitting a linear regression; the transformation improves the estimates further. However, the authors do not provide details about their models and only utilize simulated data from Aimsun (i.e., no ground-truth data) to validate their approach. The study in [17] utilizes LD data to calculate speed, traffic volume, and the LD’s occupancy. The acquired data serves to validate a developed freeway travel time estimation model with Discrete-Time Markov Chains. The model shows promising results with travel time deviations lower than 3% from the LD data. Nevertheless, a comparison to an actual empirical ground-truth measurement is not provided.

Besides the aforementioned works, research also focuses on the fusion of different data sources to achieve accurate travel time estimates, e.g., LD and probe vehicle data [18] or drone data to derive naturalistic vehicle trajectories [19]. In addition, the travel time reliability is highlighted by several kinds of research works as an essential measure to evaluate not only empirical measurements but also estimated/predicted outputs (see, [20] or [21]).

## 3. Methodology for Representing Urban Traffic States

This section presents the methodology to infer traffic flow and travel times from the available data sets throughout this work. In addition, we introduce performance metrics that are used to evaluate the derived time series.

**Figure 1:**
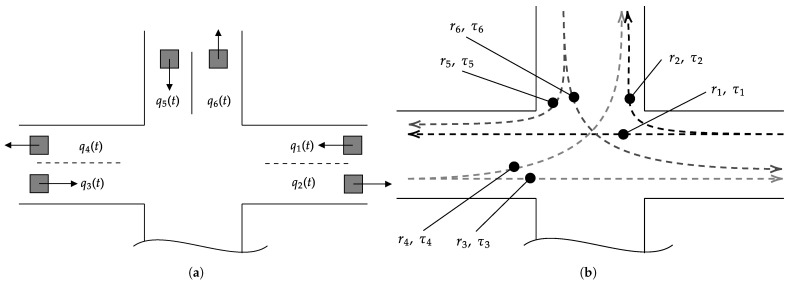
Defined quantities for derivation of traffic flow and travel time: (**a**) measurement spots to derive traffic flow $q_{s}\left(t\right)$ for all s={1,2,3,4,5,6}, (**b**) pre-defined routes r={1,2,3,4,5,6} to derive all travel times $\tau_{r}\left(t\right)$.

Let us consider a four-leg intersection system depicted in Figure 1. Note that one intersection approach located south is assumed to not provide any sensors. Hence, this approach is not considered for deriving traffic flow and travel times. For the remaining approaches, the fundamental quantities traffic flow $q_{s}\left(t\right)$ of pre-defined points *s* in space and travel time $\tau_{r}\left(t\right)$ for a pre-defined route *r* are defined. Figure 1 depicts all spots *s* and routes *r*. Note again that for the approach from the south no elements of *s* and *r* are available. Since no sensors are deployed on this road section, an in-detail investigation is not possible. Consequently, six measurement spots to derive a set of traffic flow $F=\{q_{1}\left(t\right),q_{2}\left(t\right),q_{3}\left(t\right),q_{4}\left(t\right),q_{5}\left(t\right),q_{6}\left(t\right)\}$ and a set of six routes $R=\{r_{1},r_{2},r_{3},r_{4},r_{5},r_{6}\}$ for derivation of the set $T=\{\tau_{1}\left(t\right),\tau_{2}\left(t\right),\tau_{3}\left(t\right),\tau_{4}\left(t\right),\tau_{5}\left(t\right),\tau_{6}\left(t\right)\}$, denoting the travel times are presented throughout this work.

First, we derive traffic flow in (veh/h) from a given data source (e.g., LD, traffic signal or video data). Thus, we can define $q_{s}\left(t\right)$ to derive a traffic flow time series as follows:

(1) $$q_{s}\left(t\right)=\frac{n}{T}\;,$$

where *n* represents the vehicle count, and *T* represents the measurement interval, which is constant for all sensor sources. Since the derived flow can show outliers or anomalies, a moving average of the data is derived as follows:

(2) $${\bar{q}}_{s}\left(t\right)=\frac{1}{k+1}\sum_{i=-k}^{0}q_{s}\left(t+i\right)\;,$$

where ${\bar{q}}_{s}\left(t\right)$ is the averaged traffic flow time series for *s* and *k* the window size specifying the data taken into account for the moving average of every sample of ${\bar{q}}_{s}\left(t\right)$. Note that the defined window only considers historical data; this definition helps for real-time applications, where no future values are available. If the number of available samples are less than *k* no averaged sample is computed.

To derive the travel times, we again take a given data set and derive the quantity by applying Equation (3). The time series of travel time $\tau_{r}\left(t\right)$ for a route *r* is defined as follows:

(3) $$\tau_{r}\left(t\right)=\frac{1}{N}\sum_{v=1}^{N}t_{v_{\mathrm{out}},r}-t_{v_{\mathrm{in}},r}\;,$$

where $t_{v_{\mathrm{in}},r}$ denotes the timestamp *t* of a vehicle *v* entering the system and following a route *r*. $t_{v_{\mathrm{out}},r}$ denotes the timestamp *v* finished route *r*, i.e, exits the system. To create the travel time series, we average the number of vehilces *v* denote as *N* that travel *r* at *t*. To remove outliers, the travel time series are post-processed. As $\tau_{r}\left(t\right)$ is dependent on the ${\bar{q}}_{s}\left(t\right)$, we chose to compute the weighted moving average, where ${\bar{q}}_{s}\left(t\right)$ represents the weight, i.e.,

(4) $${\bar{\tau }}_{r}\left(t\right)=\frac{1}{k+1}\sum_{i=-k}^{0}{\bar{q}}_{s}\left(i\right)\cdot \tau_{r}\left(t+i\right)\;.$$

${\bar{\tau }}_{r}\left(t\right)$ represents the weighted moving average of $\tau_{r}$.

For the sensor-based assessment, we utilize two common performance metrics to compare the data sources (for traffic flow and travel times, respectively). First, the Pearson correlation coefficient (PCC) is introduced as follows:

(5) $$\rho \left(a,b\right)=\frac{E(a,b)}{\sigma_{a}\sigma_{b}},$$

where ρ(a,b) denotes the PCC between variables *a* and *b*; E(a,b) the cross-correlation between *a* and *b*; $\sigma_{a}$ and $\sigma_{b}$ the variance of the variables, respectively. Thereby, a PCC of 1 indicates perfect postive correlation, −1 a perfect negative correlation, whereas a correlation coefficient of 0 indicates no correlation. The closer PCC to 1, the higher the correlation of the two variables [22]. Secondly, we use the mean absolute percentage error (MAPE), defined as

(6) $$\mathrm{MAPE}=\frac{1}{n}\sum_{i=1}^{n}\left|\frac{y_{i}-\hat{y_{i}}}{y_{i}}\right|\;,$$

where $y_{i}$ are the actual observations of the ground truth, and ${\hat{y}}_{i}$ is the derived traffic quantities of a data source under evaluation [23].

## 4. Travel Time Estimation Methodology

In order to evaluate the hypothesis for estimating the travel times of an origin-destination pair in an urban area with multiple data sources, we design an MLR model for estimation. The goal is to design a model that can be utilized for estimation on all available routes. We want to showcase the performance differences between two models align with the following assumptions, respectively: (a) utilization of moving sensor data (e.g. CAVs) with a penetration rate of 5% (i.e. a 5% sample of accurate real-time information is available) and (b) utilization of the 5% subset but also extracted features from LD and traffic signal data. The two models are denoted as the baseline and final model in this work. The design process therefore requires the processing of LD and signal data, and extracting relevant features serving as model inputs (see Section 4.1). We then introduce the basic definition of an MLR model, baseline model and final model in Section 4.2, Section 4.3 and Section 4.4, respectively.

### 4.1. Feature Engineering

The first data set taken into account and serving as input to the MLR model is a 5% data sample available from emulated moving sensors (e.g., probe-vehicle data, or—considering future transportation systems—CAVs that transmit, e.g., location or speed, to a centralized processing unit). Note that in our experimental campaign introduced in Section 5, these data are acquired by sampling a random 5% subset from the empirical measurements. In addition, LD and traffic signal data from the intersections’ signal controls within the investigation area are processed. A total of 48 different LDs and traffic signals are considered to extract features and compile the following complete list of predictors:

- $S_{5}$: 5% random data sample from e.g., CAVs;
- $\bar{h}$: Average headway (s) when a traffic light is green;
- $q_{I}$: Progressed flow at an intersection (veh/h);
- $\bar{o}$: Average occupancy of LDs (%);
- $\bar{p_{c}}$: Average red/green phase count (-).

To perform the training and validation of the models, the data are split into a training and test data set. A total of 70% of observations were used for training, and the last 30% for testing. For the different routes, between 76 and 82 observations were available for the training, and 33 to 35 observations to validate the estimation results (test data set).

### 4.2. Mlr Model Definition

An MLR model serves well to predict the response of a target variable; in our case, the travel time of a sample *s* and a route *r*. By defining such a model, a functional relationship is found that takes the explanatory variables as input and allows the estimation of travel times. A mathematical formulation of an MLR model can be introduced as follows:

(7) $$\begin{array}{c} {\hat{y}}_{r,s}=\beta_{r,0}+\beta_{r,1}x_{s,1}+\beta_{r,2}x_{s,2}+\ldots \\ +\beta_{r,p}x_{s,p}+E_{r,s},\;\forall s\in \left\{1,\ldots ,T\right\}\;, \end{array}$$

where ${\hat{y}}_{r,s}$ is the response or target variable, i.e., the travel time estimate for a route *r* and sample *s*. All variables denoted as β and the corresponding subscript are regression coefficients and determined during the training procedure. The intercept of the MLR equation is denoted as $\beta_{r,0}$; $\beta_{r,1}$ to $\beta_{r,p}$ denote all the coefficients for *r* and a number of *p* predictors. The predictors are deterministic variables and represent the input features derived in Section 4.1; In (7) the variables are defined as $x_{s,1}$ to $x_{s,p}$. The error term is denoted by $E_{r,s}$ and follows a Gaussian distribution and *T* denotes the estimation horizon. The definition of the model is based on works such as [24]. The solution for ${\hat{y}}_{r,s}$ is found by applying the ordinary least square method.

### 4.3. Baseline Model Specification

For evaluation, if data fusion with LD and traffic signal data improves the estimation model, a baseline model is specified. Only with the 5% data sample as a predictor is an MLR designed for all routes *r* as a baseline. This allows to compare the travel time estimates $\hat{\tau }$ of all *r* and select a baseline model for the area of investigation (the model providing the lowest error). Consequently, we can redefine (7) with only one explanatory variable and specify the baseline model as follows:

(8) $${\hat{\tau }}_{r,s}^{b}=\beta_{r,0}^{b}+\beta_{r,1}^{b}S_{5,s}+E_{r,s}^{b},\;\forall s\in \left\{1,\ldots ,T\right\}\;,$$

Note that the superscript ’b’ in (8) indicates the specification of the baseline model.

### 4.4. Final Model Specification

The final MLR model is determined by utilizing all the features presented in Section 4.1. To determine the model with best performance for all the routes *r*, an iterative process of predictor forward selection is performed to determine the lowest error (see the defined performance metrics in Section 4.5). Consequently, we can redefine (8) and specify the final model as follows:

(9) $$\begin{array}{c} \log\left({\hat{\tau }}_{r,s}^{f}\right)=\beta_{r,0}^{f}+\beta_{r,1}^{f}S_{5,s}+\beta_{r,2}^{f}\log\left({\bar{h}}_{s}\right)+\beta_{r,3}^{f}q_{I,s}+\\ +\beta_{r,4}^{f}{\bar{o}}_{s}+\beta_{r,5}^{f}\log\left({\bar{p}}_{c,s}\right)+E_{r,s}^{f},\;\forall s\in \left\{1,\ldots ,T\right\}\;, \end{array}$$

Note that (9) shows non-linear variable transformations by utilizing the log(·) function. This methodological step is performed to change the regression relation and find the model with the best performance. In addition, the response variable ${\hat{\tau }}_{r,s}^{f}$, i.e., the travel time for a route is also transformed with log(·). The superscript ’f’ in (9) indicates the specification of the final data fusion model.

### 4.5. Performance Metrics

Two performance metrics are utilized to evaluate the performance of the baseline and final determined MLR model. The coefficient of determination ($R^{2}$) expresses the percentage of the response variables’ total variation that is explainable by the predictor variables. However, higher values of the $R^{2}$ measure can occur when more predictor variables are added to the model, leading to a distorted comparison. To account for this problem, the adjusted $R^{2}$ ($\mathrm{adj}R^{2}$) is introduced. $\mathrm{adj}R^{2}$ uses a penalty term that is dependent on the number of predictor variables (*p*). Additionally, the MAPE is utilized again as a measure to evaluate the travel time estimates of the model (i.e., the estimation compared to the test data set).

## 5. Description of Experimental Campaign and Data Sources

As this study focuses on traffic state estimation via traffic flow and travel time derivation in an urban environment, a small area is selected that reflects the quantities variance but does not allow for complex traffic movements, e.g., a high number of possible routes. The particular area is located in Zurich, Switzerland, in the northern part of the city. The experimental campaign to compile data from multiple sensors and the video data is described in the following section; similarly, the various data sources utilized here for quality assessment are also introduced.

### 5.1. Experimental Campaign with Video Cameras

**Figure 2:**
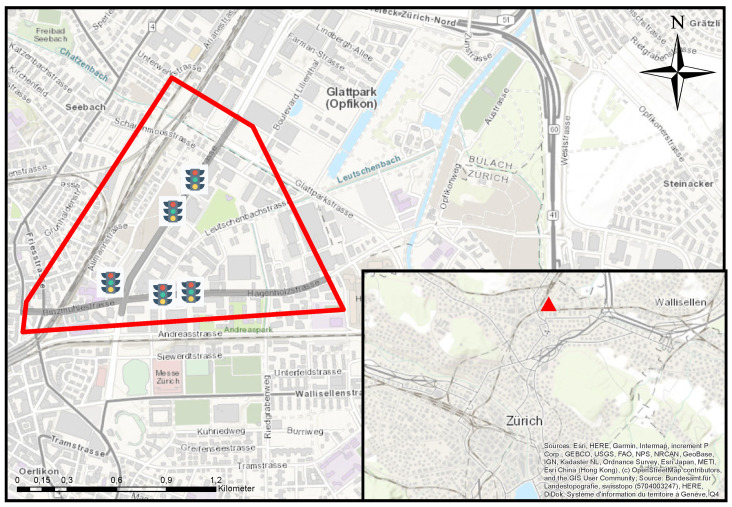
Test area (in red), Zurich Switzerland. The highlighted network in gray indicates the road segments where data is collected and processed. The traffic light symbols indicate the five implemented traffic control systems.

In Figure 2, the area under investigation is depicted in red. Additionally, the road sections that are investigated with different data sources are highlighted in gray. The approach starting in the west named Binzmühlstrasse is denoted as west bound (WB); the east approach called Hagenholzstrasse as east bound (EB); while the approach from the north named Thurgauerstrasse is given as north bound (NB). The transportation network in the red area primarily serves individual motorized transport and is regulated with five traffic control systems. A bus line operates on the west-east axis (WB to EB) and vice versa (EB to WB). Bicycle traffic is managed with cycle paths implemented on the road or with separated cycling infrastructure.

Although Zurich shows a good coverage of various sensors over the whole city, the area is selected for the following reasons: (a) the deployed sensors are spatially close to each other. Thus, the complexity is adequately low, considering the number of traffic movements allowed and the number of intersections. (b) the installed sensors allow the observation of three out of four intersection approaches. (c) the area shows good coverage with loop detector and signals control devices utilized for the feature extraction for travel time estimation.

For carrying out the empirical experiment, a prior analysis of traffic data from [25] was performed that proved the existence of morning and evening peak hours. However, the evening peak hour showed more severe congestion. Therefore, the measurement was taken in the period between 4:00 p.m. and 6:00 p.m., i.e., traffic movements are filmed for two hours on 31 March 2021. To determine the video data for all traffic flows $q_{s}\left(t\right)$ and travel times $\tau_{r}\left(t\right)$, measurements with video cameras were performed. Six HD cameras with tripods were placed according to a prior-analysis of adequate measurement spots to cover all three traffic axis in the area. The high-quality cameras used have the following specifications: two SONY HDR-PJ810 (video resolution 1920 × 1080, 24 frames per second), three SONY AX53 4K, and one SONY AX43 4K (both types with a video resolution of 3840 × 2160, 30 frames per second).

**Figure 3:**
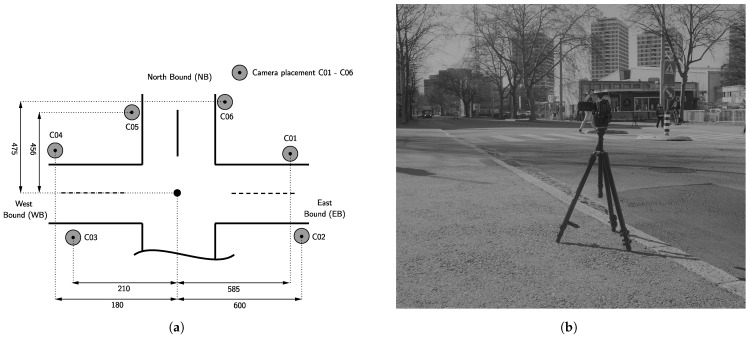
Set-up for empirical measurements: (**a**) placement and distance from the intersection center (in meters) of six cameras capturing traversing traffic (C01–C06), (**b**) used camera set-up with HD camera and tripod.

**Figure 4:**
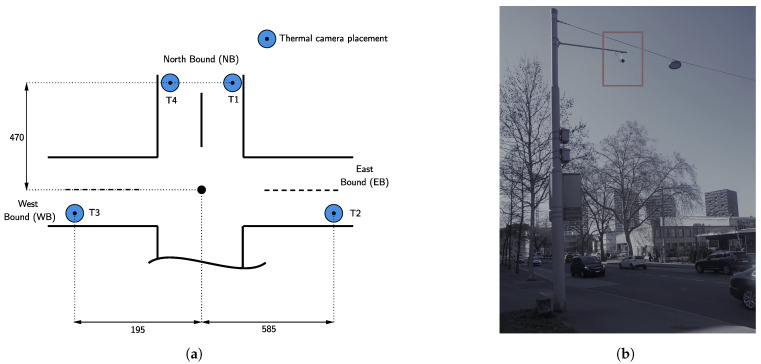
Thermal camera set-up: (**a**) four mounted thermal cameras and distances from the intersection center (in meters) capturing traversing traffic (T1–T4), (**b**) example of an overhead mounted thermal camera.

The cameras are observed by one person each to ensure a correct and precise measurement procedure. The spots C01–C06 are depicted in Figure 3a and the camera set-up in Figure 3b. For the positioning of the cameras, the following conditions were ensured: No influence of traffic flow or traffic behavior; no objects crossing the camera image such as pedestrians; a camera angle that allows minimization of disturbances due to light reflections, for instance. A camera positioning that ensures that the conditions hold allows the elimination of error sources in the automated video processing carried out later in this work. Note that the area under investigation has already equipped thermal cameras. Hence, the placement C01–C06 was chosen as close as possible to the installation spots of thermal cameras. Note that we account for small distance deviations in Figure 3 and Figure 4 in the processing described later. This ensures maximal comparability of the derived quantities.

### 5.2. Data Sources for Sensor Assessment

To develop a sensor-based analysis for traffic state representation in terms of traffic flow and travel time and provide an accuracy assessment, the following data sources are introduced in this section: already established thermal sensors in the area, processed video data with the application of an ALPR algorithm [26], a compiled data set from the Google Distance Matrix, and the manual inspection of the video data to derive the true traffic flow and travel times.

Due to several disadvantages of visual cameras (e.g., privacy concerns, lightning disturbances, visibility of objects, no observability of objects during the night), recently, thermal cameras became more popular to observe traffic. Such sensors function by capturing infrared radiation emitted by objects [27]. Although thermal cameras indicate problems of distinguishing objects of the same temperature that lie close together, the sensors outperform traditional sensors in road user classification at night while performing similarly at day time and under varying temperatures. Furthermore, it was shown that instantaneous speed was determined more accurately by thermal cameras throughout all conditions [28]. Additionally, camera sensors (visual or thermal) have also been combined with Bluetooth or WiFi sensors that allow capturing the signals of mobile devices. When a mobile device is detected, a unique MAC address can be identified that enables vehicle identification and re-identification [29]. By matching the unique MACs occurring at two or more sensor locations in a network, travel times and the average speed can be determined [30].

The area under investigation is equipped with four overhead thermal cameras as depicted in Figure 4, T1–T4. Note that the T2 and T3 capture both directions of traffic. The camera technology is capable of detecting vehicles with user-defined virtual detection zones. Thus, the determination of traffic flow for the set F is possible. Additionally, the camera detects WiFi signals which allow the derivation of travel times T in the area. For the export of the post-processed data, a commercial software system deployed by the camera manufacturer is used. This should allow practical insights into the data quality practitioners can expect when using such techniques for traffic management.

To show the capabilities of the empirical video data, we apply an ALPR algorithm to post-process the recorded frames. ALPR is the automated process of recognizing an LP and matching the correct letters and numerical characters. Different research areas have proposed a variety of open source and commercial solutions for ALPR [31]. However, such algorithms are often tested under idealized conditions (e.g.,no reflections due to sunlight, an acute angle for capturing the LP). Nevertheless, [26] proposed an algorithm based on (a) the open-source network YOLO [32] for object detection, (b) the detection of the LP with a novel proposed warped planar object detection network (WPOD-NET), (c) the LP rectification with a convolutional neural network (CNN) model and (d) the matching to letters and numerical characters with optical character recognition (OCR) by utilizing a modified YOLO network. [26] shows that the proposed method outperforms academic and commercial ALPR approaches with the processing of challenging test samples. We apply a novel algorithm from [26] to our video data set to determine traffic flow and travel times. The algorithm applies YOLO to detect objects as a first step. As we ensure that only vehicles are detected, we can use the count as a proxy to determine traffic flow. The count data is aggregated to show a flow time series of vehicles per hour. Travel times require a few more processing steps as we are the first to apply this algorithm in Switzerland (i.e. filtering different LP formats). When an LP is identified as correct by the filtering procedure, the timestamp of entering or exiting a route *r* is stored in the data set. Swiss LPs are different compared to others like European LPs. The LP shows (from left to right) the coat of arms of Switzerland, an abbreviation for the Canton (always two characters, e.g., ZH for Zurich), a number from 1 to 999999, and the coat of arms of the Canton. Consequently, the processing has to ensure that OCR recognizes a string with a correct canton abbreviation and a string that represents a number in [1, 999999]. All other detected samples are ignored. Note that the ALPR algorithm ignores symbols. Hence, no problems with the coat of arms occur. Additionally, special LPs such as the military (starts with an abbreviation “M”) are ignored by the filtering procedure (such LPs only account for a small fraction of vehicles).

Google allows the requesting of travel distance and travel time data via the Google Distance Matrix API. The API returns data based on HTTPS request information, including a start and end location (specified as GPS points). For full documentation and parameterization of the API, the interested reader is referred to [33]. In this work, we first specify the set of routes R with a start and endpoint as latitude and longitude coordinates. In addition, the heading angle of traffic is parameterized (i.e., for $r_{1}$ the angle, is equal to 90 degrees, indicating traffic moving east). Finally, we collect travel times of all routes for the transportation mode ’driving’ and the traffic model ’best_guess’. As the Google Distance API only allows to request data for the current timestamp or travel time predictions, historical data can not be accessed. Therefore, we could not obtain the data set for the time frame, we performed the empirical measurements with the video cameras. Hence, we developed an algorithm that tracks travel times for all routes every Wednesday for the identical time frame the empirical measurement was performed. Consequently, we build up a data set with Google Distance Matrix travel times of three Wednesdays. The developed algorithm sends HTTPS-requests to the Google API, post-processes the response and adds a new entry to the data set.In a situation where traffic demand in the area is similar every Wednesday from 4 p.m.–6 p.m., we compare the average of the collected travel times to the other data sources. Note that this data set only allows the determination of travel times, and that is not further processed with the methodology presented in the next section.

Finally, the video data were processed manually to compile the ground-truth data set. The two hours of video material were inspected by hand, and (a) vehicles were counted per minute, and (b) the time stamp a vehicle passes a spot *s* of every intersection approach was determined. In every recorded video, a virtual boundary was defined. When a vehicle is crossing such a boundary, the timestamp of the corresponding video frame is collected. Note that due to privacy regulations, the data set required the deletion of all LPs after the data collection, i.e., we assigned a unique ID to every vehicle in the system to determine $q_{s}\left(t\right),\;\forall s$ and $\tau_{r}\left(t\right),\;\forall r$.

## 6. Results

In this section, the sensor-based assessment of traffic flows and travel times are shown in the first subsection. The second subsection presents the estimation results of the proposed MLR models and compares performances with the defined performance metrics.

### 6.1. Traffic State Representation and Sensor-Based Assessment

**Figure 5:**
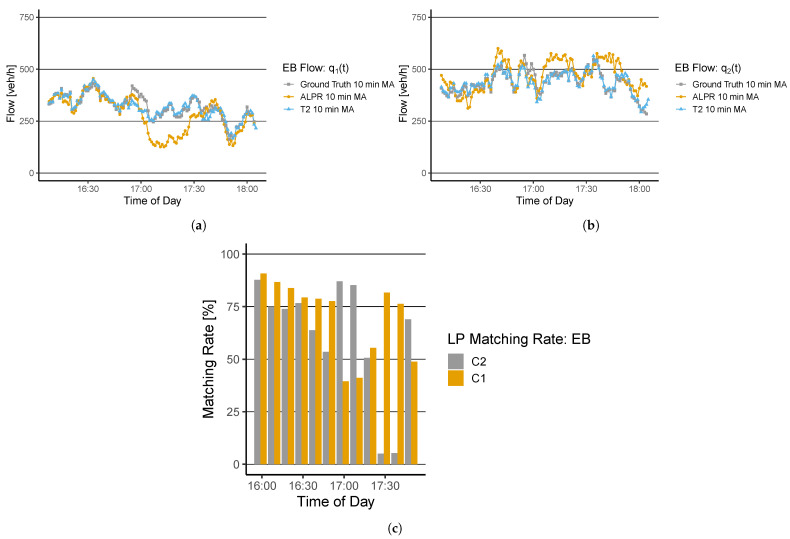
Flow evaluation of (**a**) $q_{1}\left(t\right)$ and (**b**) $q_{2}\left(t\right)$ at WB from the ground-truth measurement, the ALPR, and the thermal camera T2; (**c**) shows the matching rate of the ALPR algorithm for both derived flows.

The traffic flow results are derived by determining the vehicle counts from (a) the installed thermal cameras (b) the detected vehicles from the ALPR algorithm, and (c) the empirical measurement, i.e., the ground-truth data set. Note that the Google Distance Matrix does not allow the derivation of traffic flow. We derive all data sets and calculate the 10 min moving average of all time series $q_{1}\left(t\right)$–$q_{6}\left(t\right)$, i.e., the window size k=10. Figure 5a,b present the derived flow time series $q_{1}\left(t\right)$ and $q_{2}\left(t\right)$ for all data sets. In addition, Figure 5c denotes the matching rate of the ALPR algorithm. The matching rate is calculated by the fraction of detected vehicles by ALPR and the actually vehicle count from the ground-truth data set.

A comparison $q_{1}\left(t\right)$ and $q_{2}\left(t\right)$ of the ground-truth (in gray) and the data from the thermal cameras (in blue) T4 and T1 show a high correlation between the time series. Only small deviations of the thermal cameras’ detection are noticeable. A quantitative analysis of the correlation coefficient ρ and the MAPE show a coefficient result of 0.91 and an error rate of 4.83% for $q_{1}\left(t\right)$ and ρ=0.93, MAPE=3.33% for $q_{2}\left(t\right)$ (Table 1). Further, the ALPR results are depicted in orange. The ALPR algorithm shows a good fit for $q_{1}\left(t\right)$ until the flow drops around timestamp 17:00 significantly. This can also be seen in the matching rate of C1 that drops below 50%. After 17:30, the performance increases again with a matching rate around 70% to 75%. The average matching rate of C1 is 70%, ρ=0.81, and MAPE=15.54% (Table 1).

**Figure 6:**
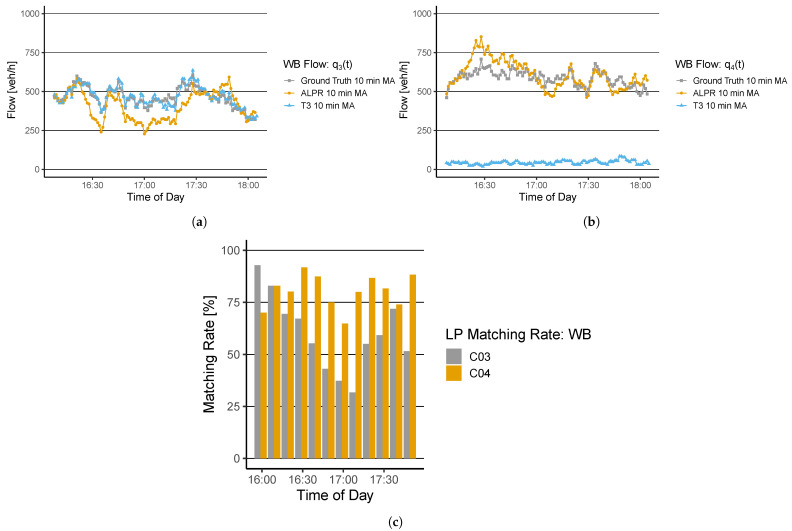
Flow evaluation (**a**) $q_{3}\left(t\right)$ and (**b**) $q_{4}\left(t\right)$ at EB from the ground-truth measurement, the ALPR, and the thermal camera T3; (**c**) shows the matching rate of the ALPR algorithm for both derived flows.

For $q_{2}\left(t\right)$, the ALPR shows a lower performance compared to $q_{1}\left(t\right)$. Several significant deviations from the ground-truth data can be observed throughout the investigated period. The matching rate of C2 shows an average rate of 59%. Nevertheless, it is observed that the matching rate around 17:30 is below 10%. The error metrics show a performance of ρ=0.61, and MAPE=12.64%. Figure 6 depicts the results for all data sources of $q_{3}\left(t\right)$ and $q_{4}\left(t\right)$, respectively.

**Figure 7:**
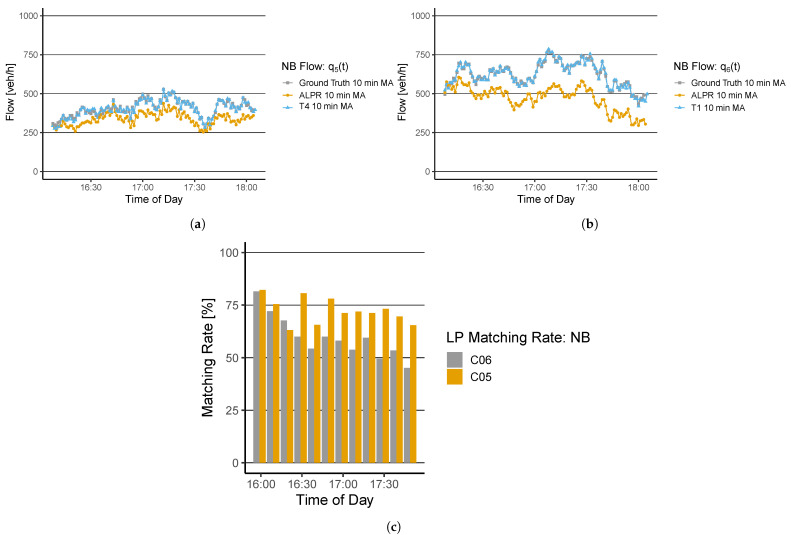
Flow evaluation (**a**) $q_{5}\left(t\right)$ and (**b**) $q_{6}\left(t\right)$ at NB from the ground-truth measurement, the ALPR, and the thermal camera T4 and T1; (**c**) shows the matching rate of the ALPR algorithm for both derived flows.

Again, a good fit of $q_{3}\left(t\right)$’s ground-truth data and the thermal camera data from T3 can be observed (ρ=0.94, MAPE=3.86%). The ALPR results show significant deviations from the ground truth over time. Especially around 16:20, a drop in the flow is observed. In addition, from 16:45 until 17:30, deviations are observed and are supported by the matching rate (Figure 6c) dropping below 50%. The average matching rate is calculated with 59%, ρ=0.58, and MAPE=17.20%. Figure 6b shows a high deviation from the ground-truth of the data set derived from T3. No correlation between the ground-truth and the thermal camera data is determined, i.e., ρ=0.00 and MAPE=92.45%. The reason for the high deviation is that T3 observes both traffic directions with one camera. Due to the high lane width (two lanes for individual transport and two additional lanes for public transportation), T3 cannot observe the traffic flow in this direction. A re-positioning of the camera or installing a second thermal camera could help improve the results. The ALPR shows a correlation of ρ=0.73 and MAPE=9.17%. The average matching rate is calculated with 76%. The performance metrics are collected in Table 1. Figure 7 shows the results for $q_{5}\left(t\right)$ and $q_{6}\left(t\right)$.

The thermal cameras of T4 and T1 show good results for $q_{5}\left(t\right)$ and $q_{6}\left(t\right)$. Note that two thermal cameras are installed due to a tram line between the two traffic lanes. For the flow $q_{5}\left(t\right)$, the correlation ρ=0.94 and MAPE=3.86%; for $q_{6}\left(t\right)$, ρ=0.99 and MAPE=1.22%. The ALPR algorithm shows deviations for both quantities. For $q_{5}\left(t\right)$, the average matching rate is 70%, ρ=0.58 and MAPE=17.20%. The results for $q_{6}\left(t\right)$ show decreased ALPR algorithm performance over time. An inspection of the ground-truth video material showed that this is caused by increasing light reflections over time. The average detection rate is equal to 57%, ρ=0.76 and MAPE=24.42%. Again, the quantitative results are collected in Table 1.

The travel time results are derived by determining the timestamp when a vehicle enters and exits the system. As data sources (a) the installed thermal cameras, (b) the detected license plates from the ALPR algorithm (c) tracked data from the Google Distance Matrix API, and (d) the empirical measurement, i.e., the ground-truth data set are used. We derive all travel time data sets and calculate the 10 min weighted moving average (the window size k=10) of the routes $r_{1}$, $r_{3}$, $r_{4}$, and $r_{5}$; consequently, $\tau_{1}\left(t\right)$, $\tau_{3}\left(t\right)$, $\tau_{4}\left(t\right)$, and $\tau_{5}\left(t\right)$. Note that for $r_{2}$ and $r_{6}$, the ground-truth data set showed low traffic volume (7 and 19 vehicles for the measurement period, respectively). Thus, data gaps occur, and a comparison of different data sources would not lead to a representative result. Therefore, these two routes are excluded from the analysis.

**Figure 8:**
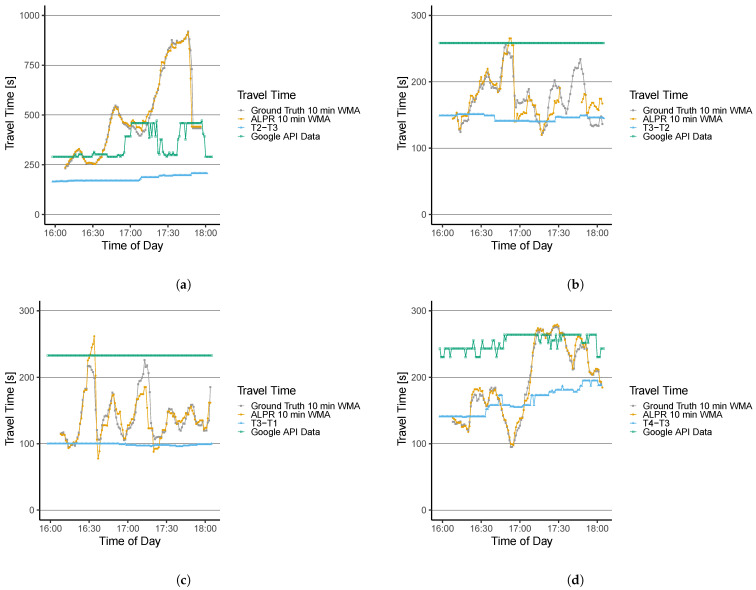
Travel time evaluation for (**a**) $\tau_{1}\left(t\right)$, (**b**) $\tau_{3}\left(t\right)$, (**c**) $\tau_{4}\left(t\right)$ and (**d**) $\tau_{5}\left(t\right)$ with the ground-truth measurement, the ALPR, the set of thermal cameras, and the Google Distance Matrix API data set.

Figure 8a,b presents the derived travel time series $\tau_{1}\left(t\right)$ and $\tau_{3}\left(t\right)$ for all data sets. The travel time results for $\tau_{4}\left(t\right)$ and $\tau_{5}\left(t\right)$ are depicted in Figure 8c,d.

The performance metrics are compiled in Table 2. One can note that the quantity $\tau_{1}\left(t\right)$ increases over time, peaks around 17:45, and decreases again afterward. Results computed from the ALPR detections (orange time series) replicate this trend with small deviations. The time series correlate with ρ=0.99 and an MAPE=3.14%. Contrary results are shown by the set of thermal cameras T2 and T3 that are utilized for travel time derivation of $\tau_{1}\left(t\right)$. The time series only shows small variations and does not capture the trend of the ground-truth data (ρ=0.71,MAPE=58.07%). Potential reasons for the modest performance can be (a) a low penetration rate, i.e., a small number of WiFi devices are detected, or (b) the data are strongly post-processed. The time series computed from the Google Distance Matrix API shows a higher variance than the thermal camera data and fails to show the variation of the ground-truth data. Especially around 17:30, when travel time continues to rise, the Google Data (green time series) does not react to this system behavior. The correlation with the ground-truth results in ρ=0.27 and the MAPE=25.95%. The results show a similar trend for the travel time results of $\tau_{3}\left(t\right)$. The ALPR results replicate the ground-truth data with some small over- and underestimations. Nevertheless, one can note a data gap from 17:30 to 17:45, where no vehicles were detected. This results in a correlation of ρ=0.84 and MAPE=8.04%. Again the time series of the thermal camera data and the Google Distance Matrix API under- and overestimate the travel time, respectively. Both methods do not allow a reaction to a travel time change from, e.g., 180 sec to 250 sec at 16:45, as the peak is not covered. Additionally, the Google data does not show any variance, i.e., the standard deviation is zero. This also does not allow the determination of a correlation coefficient. Hence, Table 2 denotes such observations with ’NA’. For the thermal camera data, the computed performance metrics are ρ=−0.11,MAPE=18.36%, and for the Google data ρ=NA,MAPE=50.64%. For $\tau_{4}\left(t\right)$, similar results as for $\tau_{3}\left(t\right)$ are derived. The ALPR algorithm allows the derivation of travel times that show a good fit to the ground-truth (ρ=0.85, MAPE=7.51%), and the thermal camera data and Google data under- and overestimate the travel time with performance metrics of ρ=−0.13,MAPE=26.76% and ρ=NA, MAPE=73.38%, respectively. For the travel times on $r_{6}$, i.e., $\tau_{6}\left(t\right)$, the ALPR algorithm allows an accurate representation of the ground-truth data with ρ=0.99 and MAPE=2.73%. The thermal camera data shows a correlation of ρ=0.72 with an MAPE=20.60% and the Google data allows the computation of ρ=0.33 and MAPE=45.49%.

### 6.2. Travel Time Estimation Assessment

**Figure 9:**
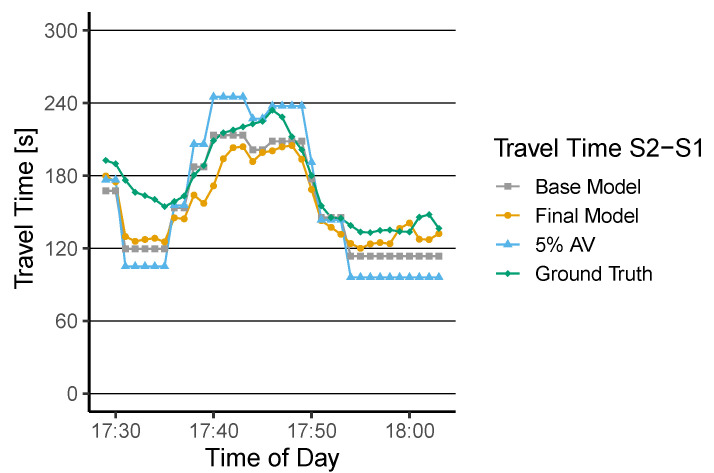
10 min WMA travel times estimates of the baseline model, estimation model, compared to the 5% data sample and the ground-truth on $r_{3}$.

To show the performance of a travel time estimation procedure, we train and apply the proposed MLR model. First, baseline models are trained with the data sample of (5%) available moving sensor data as the only predictor for all routes R. After creating the baseline, the other features are included in the model, and a final estimation model is derived. To show the performance of the trained model, we utilize $r_{3}$ as a test. Figure 9 shows the comparison of the following time series: the actual ground-truth, the 5% data sample without estimation, the travel time estimation of the baseline, and the final model.

Results show that a 5% data sample is insufficient to represent the ground-truth travel time $\tau_{3}\left(t\right)$. This is supported by a high MAPE=18.10%. The trained baseline model shows an $\mathrm{adj}R^{2}$ of 0.40 and over- and underestimates the travel time in Figure 9. The predicted time series results in an MAPE of 11.62%. Nevertheless, it can be shown that our model already improves the estimation by 6.48%. Finally, we apply the model with all features utilized as predictors. Although the model also indicates deviations from the ground-truth data, the $\mathrm{adj}R^{2}$ equals 0.81, and the MAPE reduces further to 10.92% (see Table 3).

## 7. Discussion

### 7.1. Traffic States–Traffic Flow

Traffic states were determined in terms of traffic flow and travel times in this work. Results compare first traffic flows estimated for all six measurement spots by thermal cameras, ALPR, and the ground-truth computed based on video measurements.

From thermal cameras, measurements of traffic flows with high accuracy were determined. Only time series of traffic flow $q_{4}$ show higher estimation error in comparison to ground-truth. The reason is that at this measurement spot, only one thermal camera is used to detect both traffic directions. Whereas this has worked with high accuracy for detecting flows $q_{1}$ and $q_{2}$, lane width for this intersection approach is wider due to a dedicated tram track in the center of the road. Therefore, vehicles could not be identified with the expected high accuracy. Estimation on the other traffic direction $q_{3}$ has shown low error values. This could have been caused by the mounting of the thermal camera, which is placed more on the traffic lane dedicated to $q_{3}$.

The applied ALPR algorithm also shows promising results for traffic flow. Nevertheless, some under- and overestimations could be identified, e.g., $q_{1}$ (and the corresponding camera C1), the underestimation of traffic flow corresponds to a drop in the matching rate below 45%. A manual inspection of the video measurement shows that vehicles arrive at the measurement spot in groups with short spacing. Consequently, a shorter period is available for the camera to focus, resulting in a lower detection rate of ALPR algorithm. The detection rate for C2 shows even lower values around 17:30, where the matching rate drops below 10%. Similarly, low matching rate is likely to be caused by vehicles arriving in groups. Additionally, the lane widens due to a bus stop, allowing for a vehicle position with a higher variance from the center of the lane. Consequently, situations occur where the vehicle’s license plate is covered by the front vehicle. In addition, for C3 the same situations could be identified when analyzing the video material. This causes a continuous drop in the matching rate from 16:20 to 17:10 (and therefore also deviations in traffic flow). Additionally, there is significant cycling traffic on the bike lane, which can a situation, where a cyclist traveling at a similar speed as a vehicle blocks the sight to the vehicle’s license plate.

### 7.2. Traffic States–Travel Times

For the estimation of travel times, ALPR shows a good performance in computed results. The variance of travel times shown in ground-truth data is reflected by utilizing the ALPR algorithm. Estimation errors that occur correlate with a low matching rate caused by the factors already discussed when deriving traffic flow. Note that travel time series for route $r_{3}$ show a data gap for around 15 min at 17:30. This is caused by the low matching rate that was already shown when determining $q_{2}$ with camera C2. Although thermal cameras can determine traffic flow with high accuracy, the derived travel times show high error values. The variance of our experiment’s travel times is important for short-time traffic management. Nevertheless, the thermal camera and Google Distance Matrix data do not show the real-world data trends.

Thermal cameras underestimate the travel times on all processed routes. On the other hand, Google Distance matrix data overestimate all cases. For thermal cameras, it is conjectured that penetration rate (i.e., number of vehicles that are detected via WiFi signal divided by actual number of vehicles) is low. This could be improved by additional thermal sensors upstream from the measurement points. Thus, multiple measurements could be used to infer vehicle detections and consequently increase the penetration rate. Finally, Google Distance matrix data overestimates travel times throughout the investigated period.

### 7.3. Travel Time Estimation Models

We have proposed two MLR models: a baseline model with 5% moving sensors (i.e., a random sample from ground-truth data in our case) as input and a final model with the same 5% data sample and features extracted from LD and traffic signal data. The 5% of data was chosen as an exemplary low penetration rate to consider the difficulty of monitoring traffic in an urban setting. In addition, the sample size emulates the transition period from human-driven vehicles to CAVs. The results show that the 5% data sample alone is not enough to represent the travel time of a given route (highest determined error values). The baseline model enhances the estimation, but performance could be further improved by fusing the data of extracted features (final MLR model). Although more sophisticated modeling approaches can be found in the literature for such estimation tasks, an MLR model provides a good performance baseline and also demonstrates that a methodological framework could improve travel time estimations for future mobility systems.

## 8. Conclusions

The paper presents insights on arterial roads traffic state representation in terms of traffic flows and travel times in an urban transportation network. In this work, we propose a simple yet efficient multiple linear regression (MLR) model that fuses information from several sources. The first version of MLR model, namely “baseline”, estimates travel times and traffic flows using the information of a small vehicle fleet percentage (i.e., 5%). The final MLR model fuses the above information with static loop detector (LD) and traffic signal data. For assessment purposes an experimental campaign with video measurements within a restricted area in Zurich, Switzerland has been organized, while additional data sources (thermal camera, loop detector, and signaling data) were made available to the authors by the city of Zurich.

More specifically, the work investigates a sensor-based assessment and takes into account (a) data from thermal cameras, (b) post-processed video data with an automated license plate recognition (ALPR) algorithm, and (c) travel times from the Google Distance Matrix API. All post-processed data sources are then compared to the empirical ground-truth measurements conducted in a particular urban area in Zurich, Switzerland. The traffic measurements with video cameras allow the compilation of a data set with true traffic flow of six detection spots and travel time values of six routes during two hours (afternoon peak). Results show that for the derivation of traffic flow, the thermal camera performs best with a mean absolute percentage error (MAPE) below 5% for all detection spots. The ALPR algorithm shows error values between 9% and 25%, which are due to decreasing detection rates of the procedure caused by, e.g., light reflections. For the derivation of travel times, the ALPR outperforms all other sensor technologies with an MAPE below 8% for all routes. However, the performance of data from thermal cameras and the Google Distance Matrix API is modest, with error values between 18% and 58%. Finally, we showcase the performance of the proposed MLR model for travel time estimation. The model architecture gets several extracted features from loop detector data and a 5% data sample from moving sensor data (e.g., connected and automated vehicles (CAV)) as an input. We compare the model performance with (a) the 5% data sample alone, (b) a baseline model that only sees the 5% data sample for training, and (c) the actual ground-truth data. Results show that a data sample such as 5% of the ground-truth data is not enough to represent the travel time for a specific route. With the MLR baseline model, the MAPE can be reduced from 18.10% to 11.62%; with the final model, the error reduces further to 10.92%.

## Figures and Tables

**Table 1:** Correlation coefficient ρ and MAPE of ALPR (abbreviation LP), thermal camera data (abbreviation TC) and the 10 min MA Ground Truth flow data.

	$q_{1}\left(t\right)$		$q_{2}\left(t\right)$		$q_{3}\left(t\right)$		$q_{4}\left(t\right)$		$q_{5}\left(t\right)$		$q_{6}\left(t\right)$	
	**LP**	**TC**	**LP**	**TC**	**LP**	**TC**	**LP**	**TC**	**LP**	**TC**	**LP**	**TC**
ρ [-]	0.81	0.91	0.61	0.93	0.58	0.94	0.73	0.00	0.58	0.94	0.76	0.99
MAPE [%]	15.54	4.83	12.64	3.33	17.20	3.86	9.17	92.45	17.20	3.86	24.42	1.22

**Table 2:** Correlation coefficient ρ and MAPE of ALPR (abbreviation LP), thermal camera data (abbreviation TC), Google Distance Matrix API data (abbreviation G) and the 10 min MA ground truth flow data. Note that a correlation denoted as NA=not available means that the time series standard deviation is zero.

	$\tau_{1}\left(t\right)$			$\tau_{3}\left(t\right)$			$\tau_{4}\left(t\right)$			$\tau_{5}\left(t\right)$		
	**LP**	**TC**	**G**	**LP**	**TC**	**G**	**LP**	**TC**	**G**	**LP**	**TC**	**G**
ρ [-]	0.99	0.71	0.27	0.84	−0.11	NA	0.85	−0.13	NA	0.99	0.72	0.33
MAPE [%]	3.14	58.07	25.95	8.04	18.36	50.64	7.51	26.76	73.38	2.73	20.60	45.49

**Table 3:** Adjusted R-square values of the model performance on the training data. MAPE denotes the comparison of travel times estimates (baseline model and estimation model), and 5% sample of $r_{3}$.

$r_{3}$	$\mathrm{adj}R^{2}$ [-]	MAPE [%]
5% sample	-	18.10
Base Model	0.40	11.62
Final Model	0.81	10.92

## Data Availability

Not applicable.
